# Supplementary material for: Heat Shock Protein 70 Is Involved in the Efficiency of Preconditioning with Cyclosporine A in Renal Ischemia Reperfusion Injury by Modulating Mitochondrial Functions
Source: Int J Mol Sci. 2023 May 31;24(11):9541. doi: 10.3390/ijms24119541 (PMC10253937; doi:10.3390/ijms24119541)

Supplementary data

Supplementary Figure S1. Effect of SiRNA against Hsp70 or plasmid-Hsp70 on Hsp70 protein expression of HK2 cells: Western blot analysis.

HK2 cells were transfected with a mixture of siRNA against mRNA of human HSPA1A and human HSPA1B (SiRNA HSP70, n=4 cell preparations), or a negative control siRNA (SiRNA CTL, n= 4 cell preparations), and with a plasmid containing human hspA1A/70 gene (plasmid HSP70, n=1 cell preparation) or an empty plasmid (n=1 cell preparation). Results are shown as median with interquartiles. * $p<0.05$, Mann-Whitney test.

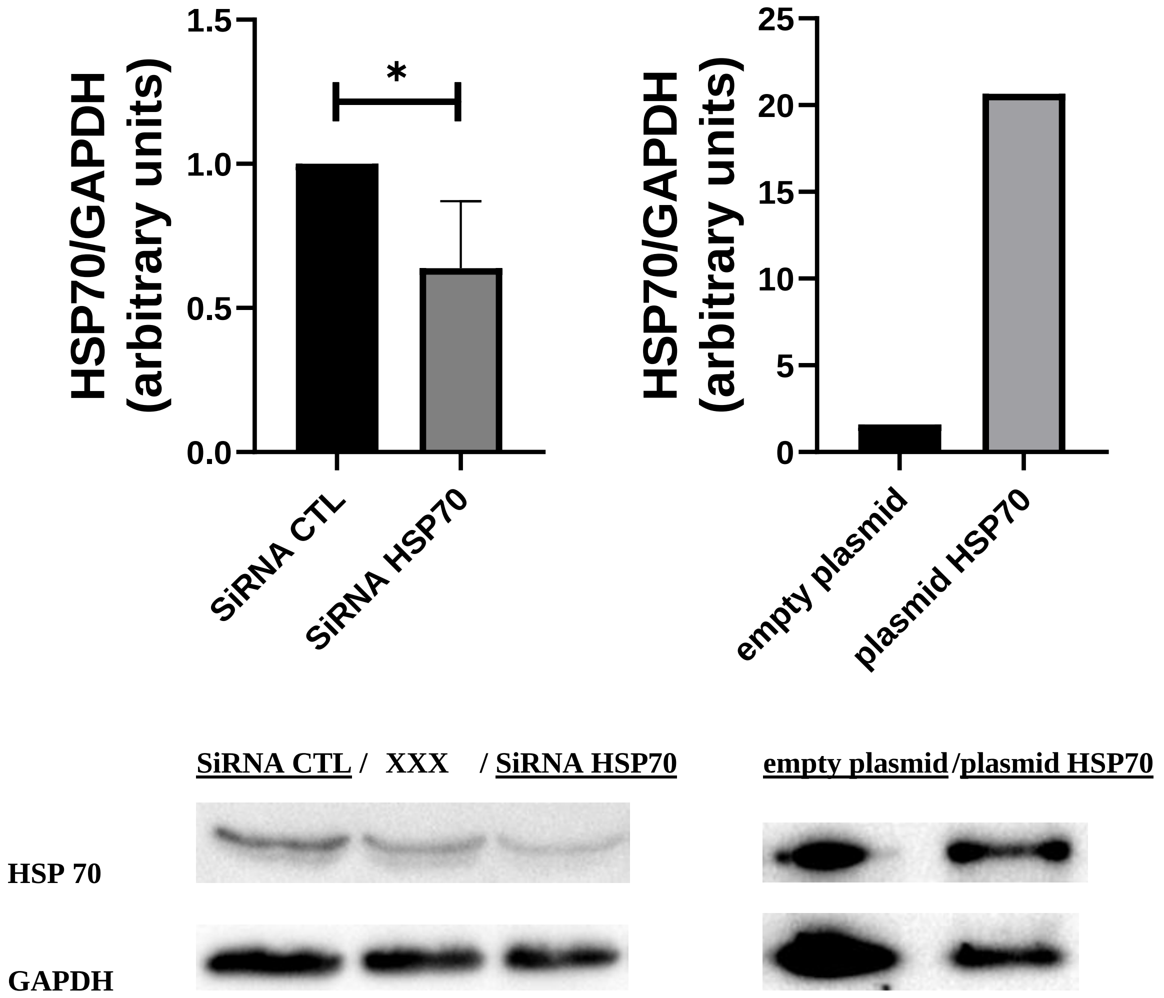

Supplementary Figure S2. Sequence of renal ischemia reperfusion in mice.

Thirty minutes of ischemia followed by 24 hours of reperfusion were performed. In the precond-CsA 10 mg/kg group, CsA was administered 10 minutes before ischemia at the dose of 10 mg/kg intravenously. In the precond-CsA 10 mg/kg + Q and the ischemic + Q groups, 100 mg/kg of Quercetin were administrated intraperitoneally 2 hours before ischemia. In the ischemic group, mice received neither CsA nor Quercetin injection. Mitochondria were isolated after 24 hours of reperfusion to assess calcium retention capacity and oxidative phosphorylation. Plasma creatinine and histological score were assessed 24 hours after perfusion. Hsp 70 immunoblotting was performed after 20 minutes and 24 hours of reperfusion.

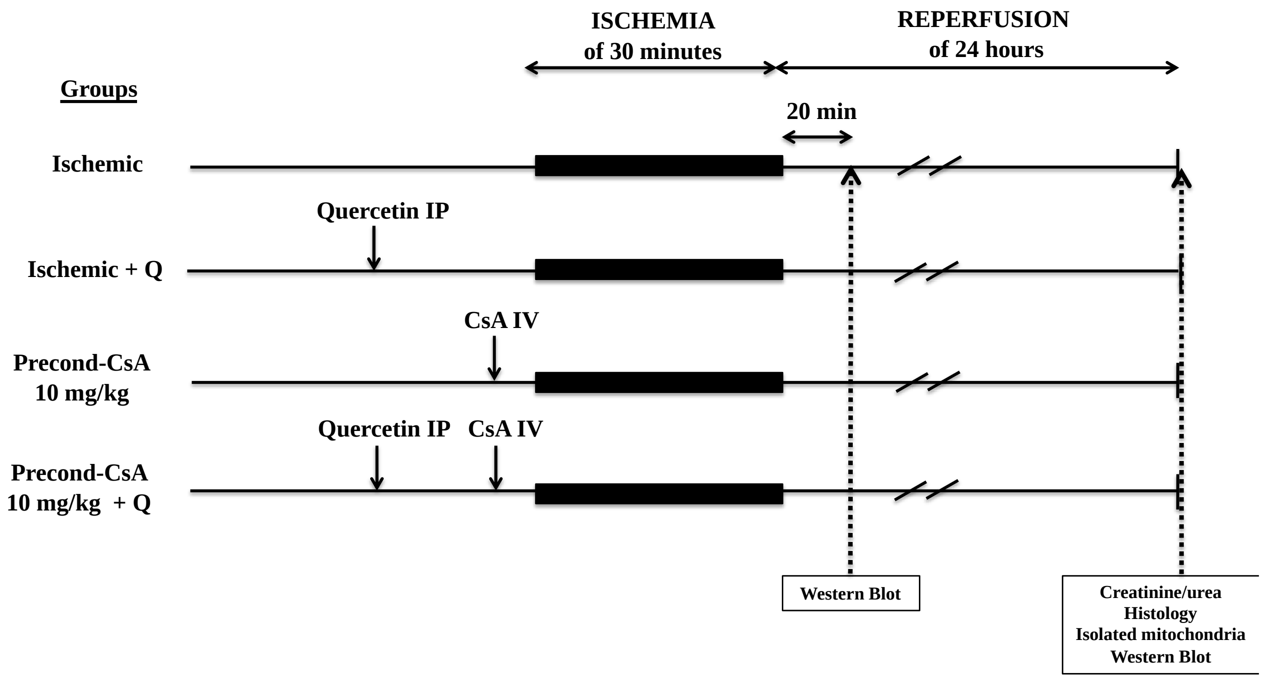

Supplementary Figure S3. Sequence of hypoxia reoxygenation on HK2 cells.

After 18 hours of hypoxia and 4 hours of reoxygenation, cells were detached from the wells to assess cell death, ΔΨm and Hsp70 immunoblotting. The effect of CsA preconditioning on HK2 cells was evaluated with an infusion of 0.5 µM of CsA for one hour, just before hypoxia. The effect of Hsp70 inhibition on HK2 cells was studied by infusing 50 µM of Quercetin for one hour, before hypoxia.

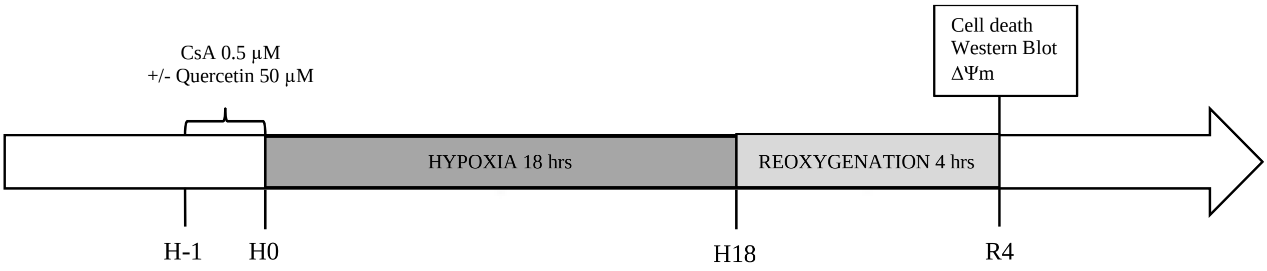

Supplement: Supplementary file 1 [file ijms-24-09541-s001.zip › ijms-2397820-supplementary.pdf]
